# Dissecting clinical outcome of porcine circovirus type 2 with in vivo derived transcriptomic signatures of host tissue responses

**DOI:** 10.1186/s12864-018-5217-5

**Published:** 2018-11-20

**Authors:** Nicolaas Van Renne, Ruifang Wei, Nathalie Pochet, Hans J. Nauwynck

**Affiliations:** 1 Laboratory of Virology, Faculty of Veterinary Medicine, Ghent University, Merelbeke, Belgium; 2 Ann Romney Center for Neurologic Diseases, Department of Neurology, Brigham and Women’s Hospital, Harvard Medical School, Boston, MA USA; 3 Broad Institute of Harvard and Massachusetts Institute of Technology, Cambridge, MA USA

**Keywords:** PCV2, PorSignDB, PMWS, STAT3, IL-2, Pathogenesis, Transcriptomics, Systems biology

## Abstract

**Background:**

Porcine Circovirus Type 2 (PCV2) is a pathogen that has the ability to cause often devastating disease manifestations in pig populations with major economic implications. How PCV2 establishes subclinical persistence and why certain individuals progress to lethal lymphoid depletion remain to be elucidated.

**Results:**

Here we present PorSignDB, a gene signature database describing in vivo porcine tissue physiology that we generated from a large compendium of in vivo transcriptional profiles and that we subsequently leveraged for deciphering the distinct physiological states underlying PCV2-affected lymph nodes. This systems genomics approach indicated that subclinical PCV2 infections suppress a myeloid leukocyte mediated immune response. However, in contrast an inflammatory myeloid cell activation is promoted in PCV2 patients with clinical manifestations. Functional genomics further uncovered STAT3 as a druggable PCV2 host factor candidate. Moreover, IL-2 supplementation of primary lymphocytes enabled ex vivo study of PCV2 replication in its target cell, the lymphoblast.

**Conclusion:**

Our systematic dissection of the mechanistic basis of PCV2 reveals that subclinical and clinical PCV2 display two diametrically opposed immunotranscriptomic recalibrations that represent distinct physiological states in vivo, which suggests a paradigm shift in this field. Finally, our PorSignDB signature database is publicly available as a community resource (http://www.vetvirology.ugent.be/PorSignDB/, included in Gene Sets from Community Contributors http://software.broadinstitute.org/gsea/msigdb/contributed_genesets.jsp) and provides systems biologists with a valuable tool for catalyzing studies of human and veterinary disease. Finally, a primary porcine lymphoblast cell culture system paves the way for unraveling the impact of host genetics on PCV2 replication.

**Electronic supplementary material:**

The online version of this article (10.1186/s12864-018-5217-5) contains supplementary material, which is available to authorized users.

## Background

Porcine circovirus type 2 (PCV2) is a very small circular single-stranded DNA virus that circulates endemically in swine populations. Its limited coding capacity of approximately 1.7 kb only allows two major viral proteins: a capsid protein (Cap), and a replication protein (Rep). An overlapping viral protein, ORF3, was found to be implicated in apoptosis, at least in vitro [1, 2]. PCV2 manifests itself through a range of often devastating pathologies in swine livestock, causing severe economic losses. The most prominent disease associated with PCV2 is post-weaning multisystemic wasting syndrome (PMWS). PMWS patients exhibit progressive weight-loss, respiratory distress, pallor of skin, digestive disorders and sometimes jaundice, coinciding with pneumonia, nephritis, hepatitis and severe lymphadenopathy. Pathologic hallmarks in wasting pigs include an elevated viral load, progressive lymphocytic depletion and monocyte infiltration in lymph nodes [3], which drastically compromises the immune system with often fatal outcome [4]. Although PCV2 is acknowledged as the causative agent of PMWS, PCV2 infection alone generally results in a persistent low-level replication without clinical signs [5]. In fact, PCV2 circulates endemically in pig populations as covert subclinical infections, seemingly undeterred by vaccination [6]. Pigs with PMWS however, are nearly always presented with concurrent microbial infections, which suggests a crucial role for superinfections in triggering PMWS [7]. Indeed, coinfections or other immunostimulations such as adjuvant administration were confirmed to produce PMWS in experimental models [8]. In a real-life setting, piglets are mostly affected after weaning. This presumably happens because maternal antibodies cease to provide protection [9]. Hence the name of the disease: PMWS.

Progress in PCV2 research is particularly hampered by the lack of tools, reagents and resources that are readily available for model species such as human or mouse. In fact, most PCV2 studies are merely descriptive and many important questions concerning its pathology remain. It is widely accepted that PCV2 can establish an asymptomatic state with low-level replication, but how PCV2 achieves such persistence is unknown [10]. Furthermore, while many studies have shown that superinfection can trigger PMWS, mechanistic insight into why certain individuals transform from subclinical PCV2 to PMWS remains unknown. For these reasons, PCV2 pathology deserves further investigation.

These days, large data sets measuring the transcriptomic architecture of biological systems are increasingly available in on-line repositories. They include those describing both clinical and subclinical infections of PCV2-affected lymphoid tissue [11, 12]. Specifically for the field of porcine biology, many individual data sets from live animals were only analyzed within the study for which they were generated. As a consequence, integrated analysis of the recent wealth of transcriptomic data opens opportunities for systems biologists. Here we take advantage of large volumes of porcine transcriptomic studies to create a novel gene signature collection of in vivo perturbation signatures. We subsequently interrogated this database against a circovirus patient study in order to better understand lymph node host responses to PCV2 viral infections.

## Results

### PorSignDB: A gene set collection characterizing a compendium of in vivo transcriptomic profiles

**Fig. 1:**
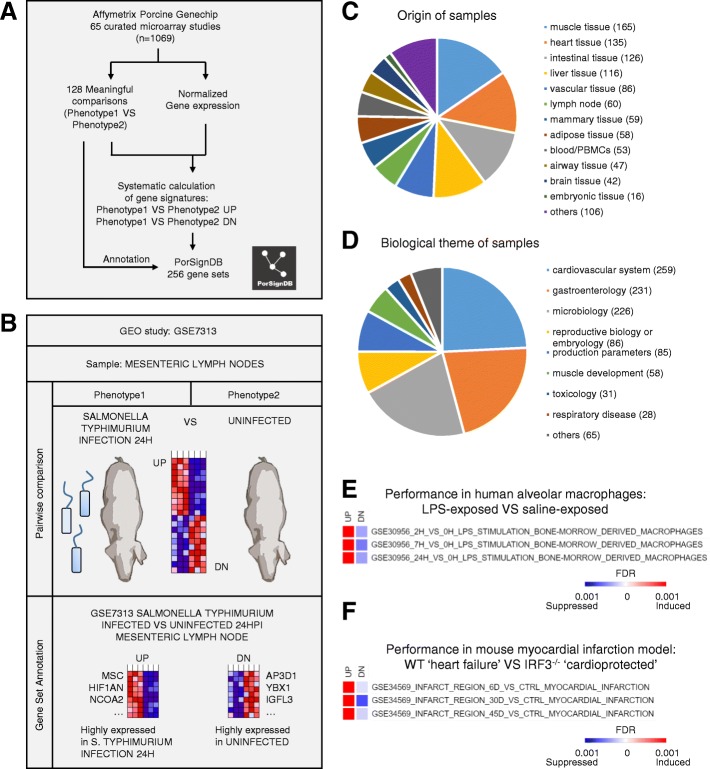
Details of PorSignDB. **a** Overview of the pipeline. 88 curated studies with data from 1776 microarrays chips were retrieved from the GEO repository. Data from each study was uniformly normalized using Genepattern, and gene expression signatures representing each phenotype of every pairwise comparison were calculated in R. Systematical annotations were added to every signature, yielding 412 gene sets. PorSignDB logo was made by NVR. **b** Example of signature generation. GSE7313 is a study mapping transcript abundance in mesenteric lymph nodes of pigs infected with *Salmonella* Typhimurium at different time points. The first pair compares data from lymph nodes of uninfected pigs (Phenotype1) with those of pigs 8 h post *S.* Typhimurium infection (Phenotype2). Significantly upregulated and downregulated genes were selected with a mutual-information based metric, respectively recapitulating highly expressed genes in the ‘uninfected’ phenotype (UP gene set), and highly expressed genes in the ‘8 h post *S.* Typhimurium infection’ phenotype (DN gene set). Clip art was made by NVR. **c** Samples were derived from a variety of different tissues, **d** covering studies in a wide range of different biological themes. **e** Performance of PorSignDB LPS gene signatures in alveolar macrophages of lungs treated with either LPS or saline solution. **f** Performance of PorSignDB myocardial infarction gene signatures in myocardial tissue of wild type VS IRF3^−/−^ knockout mice

We first created PorSignDB, a collection of porcine gene signatures, using a systematic approach previously developed for inference of the immunologic gene signature collection ImmuneSigDB [13]. Specifically, we compiled a large gene expression compendium curated from 65 studies including 1069 unique samples. A total of 256 annotated gene sets were derived from 128 pairwise comparisons identifying genes induced and repressed in one phenotype versus another, annotated as ‘UP’ (PHENOTYPE1_VS_PHENOTYPE2_UP) and ‘DOWN’ (PHENOTYPE1_VS_PHENOTYPE2_DN) gene sets, respectively (Fig. 1a). To illustrate this, an example is given for a study comparing lymph nodes of pigs experimentally infected with *Salmonella enterica* Typhimurium versus those of uninfected pigs [14]*.* Upregulated genes (UP gene set) are highly expressed in the *Salmonella*-infected phenotype, while downregulated genes (DN gene set) are highly expressed in the uninfected phenotype (Fig. 1b). Gene Ontology (GO) biological process gene enrichment was performed for every gene set, and provides an overview of the biological information captured in this signature database (Additional file 1). Gene set pairs where neither UP or DN yielded a single significant GO term enrichment hit (Benjamini-Hochberg corrected *p*-value < 0.05) were discarded in order to retain only biologically meaningful gene sets.

This approach has a number of advantages over ImmuneSigDB. First of all, ImmuneSigDB mainly covers in vitro samples. For PorSignDB however, samples were predominantly derived from real-life patients or laboratory animals (900 in vivo and 157 primary ex vivo specimens out of a total of 1069). In consequence, it constitutes a more natural description of the biological processes going on in real-life situations. In addition, while ImmuneSigDB only describes immune cell transciptomics, the scope of PorSignDB is much wider because its samples were derived from a multitude of different tissues (Fig. 1c). Together, they describe host responses in an entire range of biological themes, with a major part stemming from studies on microbiology, gastroenterology and the cardiovascular system (Fig. 1d).

Of note, porcine genes and individual probes were mapped to *Homo sapiens* ortholog genes. Because many transcriptional programs are evolutionarily conserved, cross-species gene expression analysis can be applied successfully [15, 16]. Moreover, molecular signature databases are often human-oriented, and the porcine-to-human adaptation of PorSignDB thus facilitates its application to genomic expression data of any species.

To demonstrate the validity of the information contained in the PorSignDB gene sets, we examined a study in which healthy human lungs were exposed to either lipopolysaccharide (LPS) or saline infusion in vivo [17]. In this particular study, alveolar macrophages were obtained through bronchoalveolar lavage and their transcriptomes mapped with microarray. We compared transcriptomic profiles of LPS-exposed macrophages with saline-solution exposed macrophages, and tested signatures from PorSignDB for their enrichment (induced or repressed) using Gene Set Enrichment Analysis (GSEA). Interestingly, PorSignDB also contains pairwise signatures of LPS-stimulated macrophages VS unstimulated macrophages e.g. 2H_VS_0H_LPS_STIMULATION_BONE-MORROW_DERIVED_MACROPHAGES. Indeed, PorSignDB’s gene signatures of LPS-stimulated macrophages were highly induced (Fig. 1e, UP gene sets), while the pairwise gene signatures of unstimulated macrophages were repressed (Fig. 1e, DN gene sets). This shows that PorSignDB signatures can be reproduced in comparable human datasets.

Next, we hypothesized that PorSignDB can be useful because it can label samples with the tissue-specific host-responses that they resemble. In this way, they may provide new insight into genomic data. As an example, we examined an RNA-seq dataset of a mouse myocardial infarction model. In this study, interferon regulatory factor 3 (IRF3) knockout mice (IRF3^−/−^) showed improved cardiac function and limited heart failure post myocardial infarction [18]. When comparing the myocardial transcriptomes of wild type (wt) with cardioprotective IRF3^−/−^ knockout mice in GSEA, PorSignDBs myocardial infarction tissue signatures were induced (Fig. 1f, UP), while non-infarcted healthy control heart tissue signatures were suppressed (Fig. 1f, DN). In other words, wt myocardial tissue was labeled as ‘infarcted’, while IRF3^−/−^ knockout heart tissue was identified as ‘healthy control’, corroborating their respective phenotypes. The PorSignDB myocardial infarction signatures thus provide additional evidence of IRF3 as a driver of heart failure in response to myocardial infarction. This example demonstrates that PorSignDB can be applied to any mRNA sequencing platform, and is therefore not limited to the original Affymetrix porcine system microarray from which the gene sets were derived.

Finally, the presence of multiple “viral” and “bacterial” gene signatures in PorSignDB prompted the question whether these signatures are heterogeneous, or whether they represent a single similar “infection” readout. In order to investigate this, we calculated gene overlap between bacterial and viral gene signatures (Additional file 2). This analysis shows that only minor overlap exists. This argues that the majority of viral and bacterial-related signatures represent unique readouts of host responses. Similarly, the presence of *Salmonella* Typhimurium and *Salmonella* Choleraesuis gene sets raised the question of to what extent these molecular signatures share the same information. However, gene overlap through hypergeometric test did not yield any significant hit (Benjamini-Hochberg corrected *p*-value < 0.05) (Additional file 3), indicating that there is little redundancy between the *Salmonella* Typhimurium and Choleraesuis gene sets.

The PorSignDB gene signatures are available as an online resource (http://www.vetvirology.ugent.be/PorSignDB/; Additional files 4 and 5) and can be used by systems biologists to deconvolute cellular circuitry in health and disease. As proof of concept, we employed this gene signature collection describing host responses in a wide variety of tissues to generate new insights in the multisystemic disease associated with PCV2.

### PorSignDB reveals diametrically opposed physiological states in vivo in subclinical PCV2 and PMWS

**Fig. 2:**
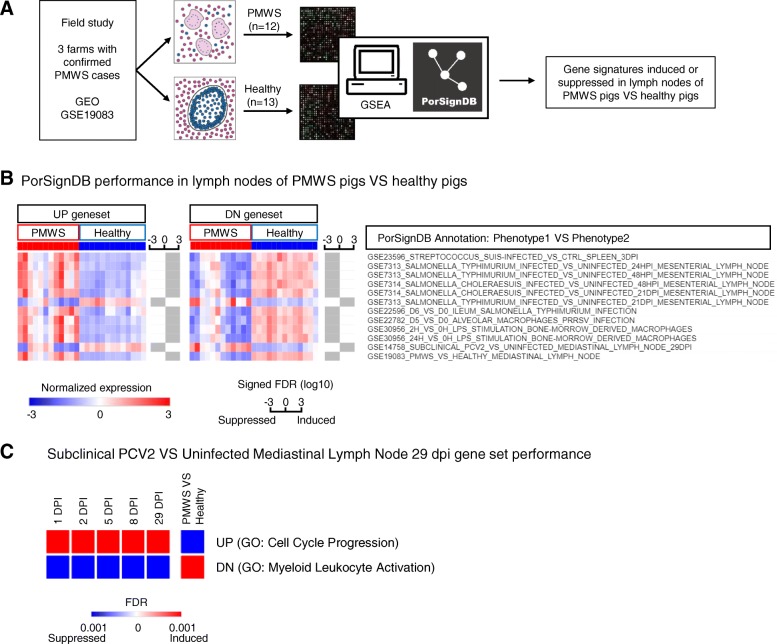
Application of PorSignDB to lymph node data originating from pig farms with naturally occurring PMWS. **a** Outline of the analysis. Data from PMWS-affected farms were retrieved from GEO. In PMWS lymph nodes, follicular structures become indistinct and B-cells and T-cells all but disappear, while infiltrating macrophages fuse into multi-nucleated giant cells. In PCV2-positive healthy lymph nodes, lymphoid structure is intact. Comparing transcriptomes of both phenotypes using GSEA displays enrichment of PorSignDB transcriptional signatures. Clip art was made by NVR. **b** Microbiology-related PorSignDB gene set expression in lymph nodes of PMWS pigs versus Healthy pigs (FDR < 0.01 and opposite expression of each pairwise phenotype). The average expression of the leading-edge genes in every gene set (genes that contribute to the enrichment) are displayed for each patient sample. Bars next to each gene set indicate the signed FDR for its enrichment in log10 scale. **c** Temporal performance of PorSignDB’s Subclinical PCV2 29 dpi infection signatures in subclinically infected pigs

We then leveraged PorSignDB to analyze a field study of pigs naturally affected with PMWS [11]. To compare transcriptomic profiles of PMWS lymph nodes with PCV2-positive but otherwise healthy lymph nodes, we tested signatures from PorSignDB for their enrichment (induced or repressed) in both classes using GSEA (Fig. 2a). We primarily focused on gene sets pertaining to microbiology. For robustness, we only retained signatures from pairwise comparisons in case both upregulated (PHENOTYPE1_VS_PHENOTYPE2_UP) and downregulated (PHENOTYPE1_VS_PHENOTYPE2_DN) genes are significantly enriched (False discovery rate; FDR < 0.01). For example, UP genes in splenic tissue of “*Streptococcus suis*-infected pigs VS control pigs” are induced (Fig 2b, left heatmap first row), while DN genes are suppressed (Fig. 2b, right heatmap first row).

Overall, this analysis reveals that upregulated genes in “microbial challenge VS control” are induced while downregulated genes are suppressed. In other words, PMWS lymph nodes display transcriptomic reprogramming consistent with tissue responses on infectious agents. This observation is supported by previous findings that naturally occurring PMWS is presented with concurrent infections [7]. Strikingly, two genomic infection signatures do not follow this pattern. First, the opposite behavior of the gene signature from *Salmonella* Typhimurium 21 days post inoculation (dpi) suggests that the *Salmonella* infection has already been cleared at this timepoint. This is indeed the case: at 21dpi the bacterial load in these mesenteric lymph nodes was reduced to undetectable levels [19]. In contrast, *S.* Choleraesuis infection was sustained at 21dpi, coinciding with persistent high bacterium abundance in mesenteric lymph nodes. Intriguingly, the second deviating gene signature originates from pigs that were subclinically infected with PCV2 (Fig. 2a, arrow). Unlike *S.* Typhimurium, this cannot be explained by pathogen clearance since these experimentally PCV2-infected pigs remained viremic throughout the original study [12]. Instead, pathogen-distressed host responses appear here to be repressed in lymph nodes with low-level subclinical PCV2 replication. Hence, highly expressed genes in “subclinical PCV2-infected VS uninfected” lymph nodes are suppressed, while lowly expressed genes are induced. Finally, the gene sets PMWS_VS_HEALTHY_UP and PMWS_VS_HEALTHY_DN serve as positive control since they were derived from the data that was queried in this instance. PorSignDB signatures from other biological themes may provide additional clues into the alterations in lymph nodes that are subject to PMWS and could be explored further (Additional file 6, see also discussion).

Interestingly, the GO analysis of PorSignDB gene sets reveals that the subclinical PCV2 infection signature 29 dpi (UP) constitutes a transcriptional program implicated in cell cycle progression (Additional file 1, gene set 33). On the other hand, the uninfected pairwise signature (DN) summarizes myeloid leukocyte activation implicated in the immune response (Additional file 1, gene set 34). In other words, this analysis suggests that upon PCV2 subclinical infection, cell cycle progression is promoted, while myeloid leukocyte immune responses are suppressed. To confirm these findings, these gene sets were interrogated in lymph nodes of pigs of the same study, but at other time points [12]. Intriguingly, the onset of both the induction of UP (GO enrichment: “Cell cycle progression”) as the suppression of DN (GO: “Myeloid leukocyte activation”) was immediate, robust, and persisted throughout all time points (all FDRs < 0.001; Fig. 2c). It should be noted that the gene signatures were derived from the 29 DPI time point, which thus serves as a positive control. We recall from Fig. 2b that this runs counter to PMWS patients, where UP is repressed and DN is induced (both FDRs < 0.001).

From this data, it can be concluded that subclinical PCV2 infection simulates pathogen-free tissue, upregulates cell cycle regulator genes and represses myeloid leukocyte activation genes implicated in the immune response. Moreover, these biological processes are reversed in PMWS patients where cell cycle genes are suppressed and myeloid cell activation is induced.

### A myeloid leukocyte mediated immune response signature predicts clinical outcome of PCV2

**Fig. 3:**
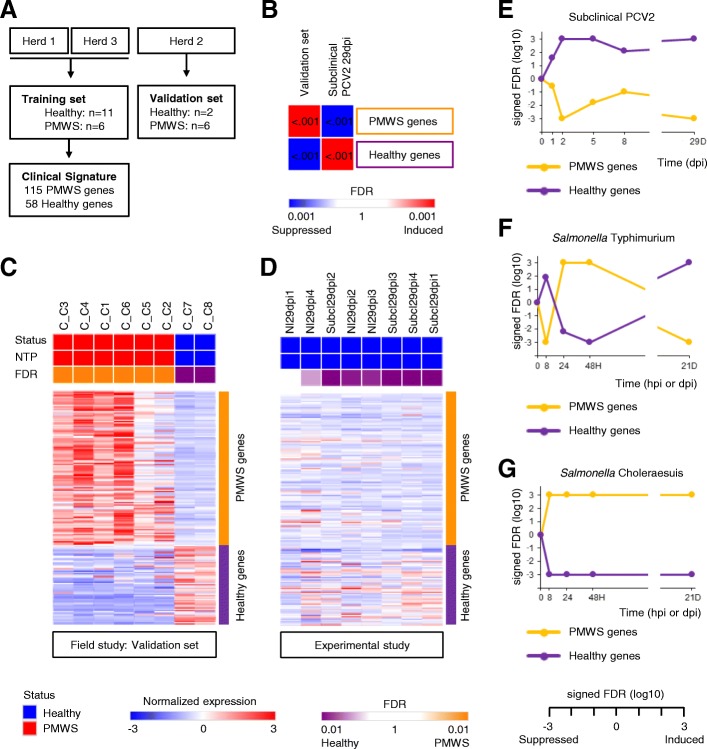
A patient-derived immune response signature predicts clinical outcome of PCV2 infection. **a** Diagram of cohort division between training and test set. A clinical PCV2 signature was calculated from the training samples and (**b**) tested in the validation samples by GSEA. The PCV2 disease signature was markedly induced in the validation set, and repressed in subclinical PCV2 29dpi. **c** Nearest Template Prediction of test set samples, classifying them either as healthy (blue) or PMWS (red), and (**d**) similarly, of the experimental subclinical infection samples at 29dpi. **e–g** Kinetics of the PCV2 disease signature upon experimental PCV2, *S.* Typhimurium and *S.* Choleraesuis infection

In an experimental setting, PCV2 alone does not lead to clinical signs. Additional superinfections or vaccination challenges are needed to produce PMWS [8]. Why extraneous immunostimulations trigger PMWS remains however poorly understood. A systems-level dissection of PCV2-affected lymphoid tissue may provide an explanation to this conundrum because it can determine which transcripts characterize PMWS, unbiased by previous knowledge. To this extent, the PMWS field study data was divided over a training and validation cohort, and 173 biomarker genes were selected from the training set using a leave-one-out cross validation (Fig. 3a, Additional file 7). Together, they reveal a molecular portrait of PCV2-associated lymphoid lesions. This ‘PCV2 disease signature’ is greatly induced in the validation cohort as shown by GSEA analysis, meaning upregulation of PMWS marker genes and downregulation of Healthy marker genes (Fig. 3b). Interestingly, in mediastinal lymph nodes with subclinical PCV2 at 29dpi, the disease signature is dramatically repressed when compared to lymph nodes of non-infected counterparts. This shows once more that in subclinical PCV2 the transcriptomic recalibration that goes hand in hand with PMWS is suppressed. To illustrate the fidelity of the PCV2 disease signature, individual samples were classified as either PMWS or healthy with the Nearest Template Prediction algorithm [20]. All samples of the validation set were correctly assigned (FDR < 0.05; Fig. 3c). Furthermore, all piglets from the experimental study, either PCV2 free or with subclinical PCV2, were correctly classified as Healthy. Only one sample failed to meet the < 0.05 FDR threshold (Fig. 3d). Furthermore, a Gene Ontology overrepresentation test indicated that the PMWS biomarker genes represent inflammatory responses and myeloid leukocyte immune activation (Additional file 8, Figure A). Of note, this gene signature performs better than an RNMI-based signature (Additional file 8, Figure B-C), which is more suited for small sample sizes and was therefore applied for generating PorSignDB.

Interestingly, when probing the kinetics of the PCV2 disease signature in lymph nodes of pigs experimentally infected with PCV2, *S.* Typhimurium or *S.* Choleraesuis, it is clear that these two bacterial infections promote the disease signature. In contrast, in subclinical PCV2 it is consistently suppressed (Fig. 3e-g). In *S.* Typhimurium the reversal of this clinical gene signature at 21 dpi coincides with the drop of bacterial load in the mesenteric lymph nodes to almost undetectable degree. This demonstrates from a systems-approach that the infection has been virtually cleared at this time point, unlike mesenteric lymph nodes upon *S.* Choleraesuis infection. In the latter, the persistence of the signature correlates with an enduring high bacterial lymph node colonization [19].

Taken together, PCV2-induced lymphoid depletion and granulomatous inflammation in PMWS patients can be summarized in a robust gene expression signature emblematic of myeloid leukocyte activation. This systems level analysis suggests that the initiation of a myeloid leukocyte mediated immune response is a pivotal event in the progression from subclinical PCV2 to PMWS.

### Functional genomics identify regulatory networks perturbations in PCV2 disease

**Fig. 4:**
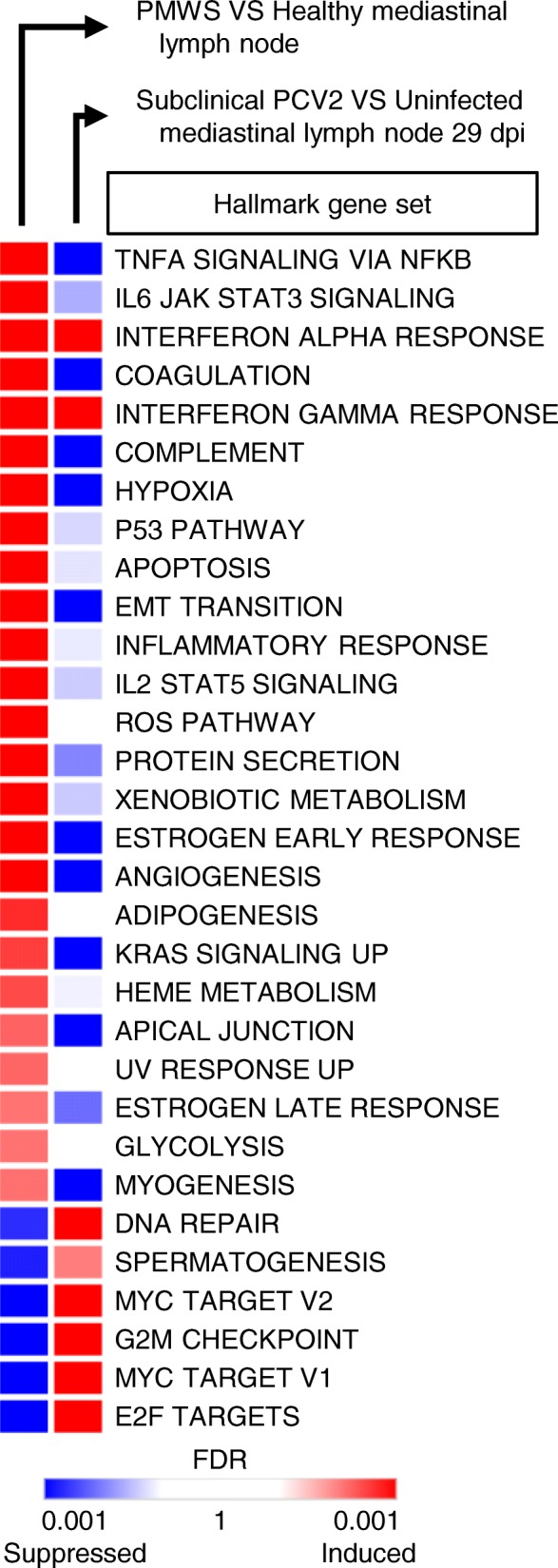
Functional genetic networks of the Hallmark gene set collection that are markedly altered in lymph nodes of pigs with PCV2. Left column: expression level in lymph nodes of PMWS patients (FDR < 0.01). Right column: expression-level of these biological circuits in Subclinical PCV2 at 29dpi

It is becoming increasingly clear that PMWS and subclinical PCV2 represent two opposing adaptations of lymphoid tissue to circoviral infection. To understand how this tiny virus arranges this *tour de force*, the data sets covering both the PMWS field study [11] and the experimentally induced subclinical PCV2 at 29 dpi [12] were interrogated in the GSEA computational system with the innovative Hallmark gene set collection [21]. This provides a very sensitive overview of alterations in a number of key regulatory networks and signaling pathways in both PMWS patients (Fig. 4a, leftmost column) and in pigs with persistent subclinical PCV2 (Fig. 4, second column). Since the molecular pathogenesis of PCV2 remains to this day mostly unexplored [10, 22], this may uncover several previously unknown network modifications [10, 22]. In lymphoid tissue of pigs with PMWS, many of the affected transcriptional networks echo key events in PCV2-associated lymphopathology such as blatant inflammatory activity (Hallmark gene set ‘Inflammatory response’) and caspase-mediated cell death (‘Apoptosis’). Increases in gene expression mediated by p53 (‘p53 pathways), reactive oxygen species (‘ROS pathway’) and NF-κB (‘TNFα signaling through NFκB’) reflect findings that PCV2 promotes p53 expression [1, 2] and triggers NFκB activation through ROS [23, 24] (Fig. 4, left column). Previously unidentified altered networks [10, 22] include immunological programs (‘Interferon alpha response’ and ‘Interferon gamma response’), cell signaling cascades (‘IL2-STAT5 signaling’, ‘IL6-JAK-STAT3 signaling’, ‘KRAS signaling up’) and bioenergetics (‘Glycolysis’ and ‘Hypoxia’).

Consistent with previous results, subclinical PCV2 infection generally fails to reproduce the imbalances associated with PMWS. Only the transcriptomic programs downstream of interferon-α and interferon-γ are in line with subclinical infections, suggesting a direct viral effect on these immunological networks. It should also be noted that the ‘Hallmark G2M checkpoint’, which describes a transcriptional cell cycle program, is induced in subclinical PCV2, and repressed in PMWS patients. This corroborates the earlier finding that genes implicated in cell cycle progression are upregulated upon subclinical infection, but downregulated in PMWS patients (Fig. 2c).

Most programs are however unaffected or opposed to the changes occurring in PMWS, reaffirming the running thread that subclinical PCV2 and PMWS represent two opposed transcriptomic recalibrations of lymph node tissue.

### IL-2 supplementation enables ex vivo modelling of PCV2 in primary porcine lymphoblasts

**Fig. 5:**
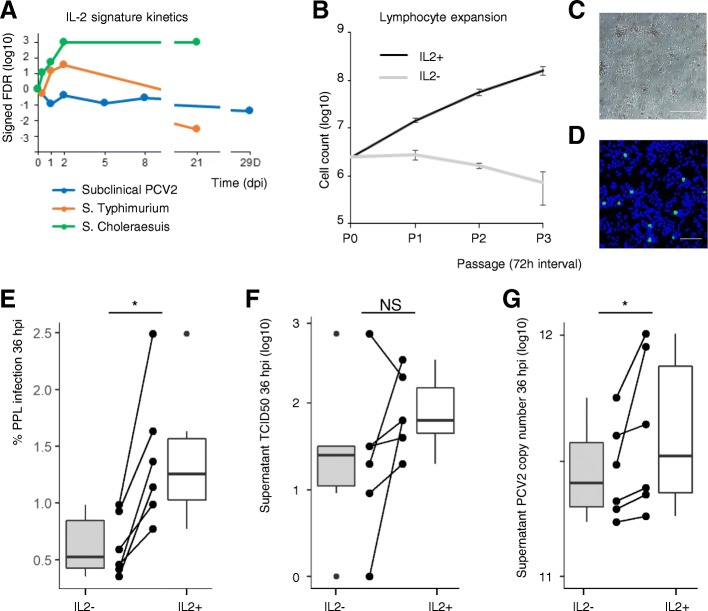
IL-2 is implicated in PCV2 disease. **a** Kinetics of IL-2 responsive gene expression (Hallmark IL2-STAT5 SIGNALING) upon three microbial infections: PCV2 (blue), *S.* Typhimurium (orange) and *S.* Choleraesuis (green). **b** IL-2 activation of freshly isolated and ConA-stimulated lymphocytes maintains exponential cellular proliferation, yielding primary porcine lymphoblast (PPL) cell strains. Means ± sd represent one experiment in triplicate (*n* = 3). **c** Representative image of proliferating PLLs. Scale bar: 50 μm. **d** PCV2 Cap immunostaining in PLLs 36 hpi. Scale bar: 100 μm. **e-g** IL-2 supplementation doubles PCV2 infection after a single round of replication (36 hpi) and increases viral load in cell supernatant. Dot blot shows six single independent experiments, box plots show the median, the 25th and 75th percentiles with whiskers representing median ± 1.5 times interquartile range (*n* = 6; *P < 0.05, two-tailed Wilcoxon signed-rank test). PPL cell strains were generated from six different individuals

An increase in viral load in lymphoid tissue is a key characteristic of PMWS [3]. In the PMWS field study, PCV2 copy number was also significantly higher in the PMWS lymph nodes compared to their healthy counterparts as measured by qPCR and in situ hybridization [11]. The Hallmark analysis therefore shows that an increase in the amount of PCV2 occurs in an environment where IL-2 responsive genes are upregulated (Fig. 4a). Given the pivotal role of IL-2 in activated T-cells during immune response [25], IL-2 may indeed be a crucial factor in boosting subclinical PCV2 towards PMWS. Intriguingly, the IL2-STAT5 signaling network is suppressed in subclinical PCV2, but not in *S.* Choleraesuis and *S.* Typhimurium, where there is a persistent and transient induction respectively (Fig. 5a). Again, in *S.* Typhimurium, the reversal of the IL-2 signature coincides with bacterial clearance.

The impact of IL-2 on PCV2 replication cannot be faithfully demonstrated with traditional PK15 kidney cells. Because PCV2 has a tropism for lymphoblasts, these are the cells of choice. Our lab previously demonstrated that treatment of freshly harvested PBMCs with concanavalin A (ConA) coerces T-cells into mitosis, rendering them permissive for PCV2 [26]. Unfortunately, lymphoblast proliferation can only be maintained for a very short time after which the cells forfeit viability and die of attrition. Indeed, when isolated lymphocytes are stimulated with ConA without IL-2, these cells start suffering from apoptosis even before the first passage at 72 h. However, supplementing ConA-stimulated lymphocytes with IL-2 generates continuously expanding primary porcine lymphoblasts (PPLs; Fig. 5b, c). These PPLs can be easily cultured, expanded and infected with PCV2 ex vivo, providing a bona fide target cell culture platform amenable for studying the PCV2 life cycle (Fig. 5d). To prove the beneficial effect of IL-2 on PCV2 replication, lymphocytes were freshly harvested from six individual pigs. IL-2 supplementation doubled PCV2 infection rates after 36 h, a timeframe amounting to a single round of replication (Fig. 5e). PCV2 titers in 5 out of 6 supernatants showed an increase upon IL-2 stimulation. A more sensitive method, measuring PCV2 genome copy numbers in cell culture supernatants showed a significant increase upon IL-2 stimulation for all 6 lymphoblast cell strains (Fig. 5f, g).

### STAT3 is a PCV2 host factor and a target for antiviral intervention

**Fig. 6:**
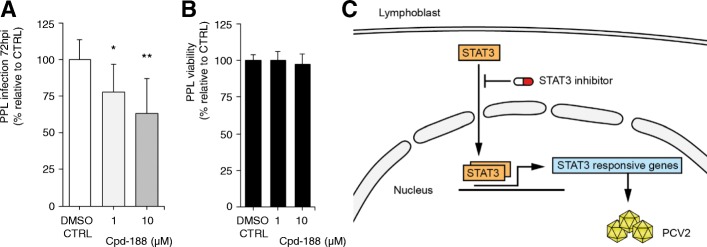
STAT3 is a PCV2 host factor. **a** STAT3-specific inhibitor Cpd188 impairs infection in PPLs. Means ± sd represent three independent experiments in triplicate (*n* = 9; *P < 0.05, **P < 0.01, two-tailed Mann-Whitney). **b** MTT lymphoblast viability assay of Cpd188 treatment. Means ± sd are shown for three experiments in quintuplicate (*n* = 15). **c** Cartoon outlining STAT3 as a drugable host factor for PCV2 in lymphoblasts. Clip art made by NVR

Since transcriptional networks of PMWS lymphoid tissue are subject to dramatic changes that correlate with fulminant PCV2 replication, counteracting these alterations can potentially harm the viral life cycle. Given the fierce induction of gene expression downstream the IL6-JAK-STAT3 signaling cascade in PCV2 patients (Additional file 9, Figure A), STAT3 emerges as a druggable candidate host factor. Interestingly, STAT3 is a key regulator of inflammation often exploited by viruses with pathogenic consequences [27]. In a drug assay, treatment with selective STAT3 inhibitor Cpd188 exhibits a dose-dependent effect on PCV2 infection in PPLs at 72 hpi (Fig. 6a). Cell viability assay reveals no toxicity, excluding non-specific adverse effects of the compound on infection (Fig. 6b). Chemical inhibition also displays a dose-dependent effect on PCV2 infection in PK15 cells (Additional file 9, Figure B-D). Thus, robust expression of STAT3 responsive genes are critical for PCV2, and hampering STAT3 activity represents an antiviral strategy (Fig. 6c).

### A paracrine macrophage-lymphoblast communication axis exacerbates PCV2 infection

**Fig. 7:**
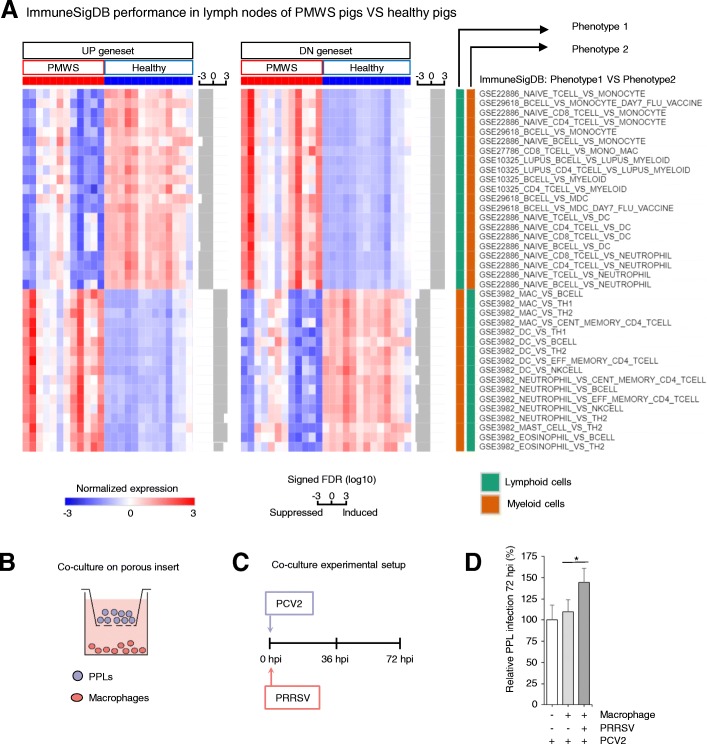
Superinfection increases PCV2 replication through a macrophage-lymphoblast paracrine signaling axis. **a** ImmuneSigDB gene set expression in the PMWS field study (FDR < 0.01 and opposite expression of each pairwise phenotype). The average expression of the leading-edge genes in every gene set (genes that contribute to the enrichment) are displayed for each patient sample. Bars next to each gene set indicate the signed FDR for its enrichment in log10 scale. PMWS versus healthy lymph node comparison displays a dramatic repression of lymphocyte gene expression signatures, and induction of myeloid cell signatures. **b** Experimental set-up of PPL-macrophage co-culture system mimicking PMWS lymph nodes. **c** PCV2-inoculated PPLs were seeded on a porous insert with macrophages at the bottom of the well. Macrophages were additionally challenged with PRRSV at 0 h. **d** Relative PPL infection levels at 72 hpi. Means ± sd represent two independent experiments in triplicate (n = 6; *P < 0.05, two-tailed Mann-Whitney)

Finally, the PMWS field study dataset (Fig. 2a) [11] was queried in GSEA with ImmuneSigDB’s immunological gene signatures [13]. At first glance, this approach may seem incompatible as ImmuneSigDB describes single types of immune cells, while the PMWS data set covers complex lymph node tissues made out of multiple cells type. However, the main constituent of lymph nodes are immune cells, which are particularly affected by PMWS. It was therefore assumed that analyzing these data with ImmuneSigDB could yield valuable information on the biological processes going on inside these lymphoid organs. Indeed, when comparing PMWS lymph nodes with healthy lymph nodes in a GSEA analysis, it revealed a striking suppression of lymphocyte gene expression and powerful induction of signatures from monocytes and other myeloid cells (Fig. 7a, Additional file 10). This reflects the loss of lymphocytes and histiocytic replacement in PMWS lymph nodes. Together with the previous observation that a myeloid leukocyte activation signature can predict clinical outcome of PCV2 (Fig. 3), it raises the question to what extent infiltrating monocytes affect PCV2 replication. After maturation into macrophages, they may either dampen infection by destroying viral particles, or promote PCV2 in a paracrine fashion by releasing pro-inflammatory cytokines. To test the effect of intercellular communication between macrophages and lymphocytes, a co-culture experiment was set up. PCV2-infected PPLs were seeded in a porous insert, physically separated from a lower compartment with primary porcine macrophages (Fig. 7b). The latter were challenged with Porcine Reproductive and Respiratory Syndrome Virus (PRRSV), a virus that can experimentally trigger PMWS [8] (Fig. 7c).

The presence of non-infected macrophages had no significant effect on PCV2 lymphoblast infection levels, but when co-cultured with PRRSV-infected macrophages, a significant and consistent increase in PCV2 infection could be discerned (Fig. 7d). Importantly, PRRSV has an exclusive tropism for macrophages [28, 29], and cannot infect lymphoblasts (Additional file 11). This excludes an effect of secondary infection of PRRSV on PCV2 replication in these lymphoblasts. This experiment thus demonstrates the existence of a previously unknown axis of intercellular communication between macrophages and lymphoblasts exacerbating PCV2 replication.

## Discussion

These days, online repositories provide an ever growing library of transcriptomic data. In this study we unlock the potential of porcine microarray studies by turning them into an atlas of transcriptional host responses on the tissue level. This approach extends MSigDB with in vivo derived profiles [30]. A considerable part of the PorSignDB gene set collection was only scantly discussed (Additional file 12), but contains interesting gene sets nonetheless. For example, the gene sets covering cystic fibrosis airway tissue may help in preclinical drug discovery by examining whether a farmacological intervention induces a ‘healthy’ signature. If in a particular transcriptomic analysis, the gene sets covering “SSEA1-NEG_VS_SSEA1-POS_FETAL_FIBROBLASTS” are overexpressed, it may indicate that SSEA1 (also known as CD15 or FUT4) is implicated in the biological process leading to the transcriptomic readout. Similarly, if gene sets describing resveratrol or deoxynivalenol-supplemented tissues are induced, it may indicate that these compounds can induce the transcriptional reprogramming that was originally queried. These are just a few hypothetical examples that illustrate the potential of these gene sets for generating hypotheses. In any case, their validity remains to be confirmed by future studies.

PorSignDB is especially convenient for delineating which physiological state one’s samples of interest resemble, generating useful hypotheses in the process. When applied to PCV2 patient data, PorSignDB shows that lymph nodes of PMWS pigs resemble those from pigs with microbial infections. At the same time, it points out that subclinical PCV2 and PMWS are two different host reactions to PCV2. It is important to discriminate between these two phenotypes of ‘PCV2 infection’, because treating them as a single entity will only result in conflicting data. As an example, this integrative transcriptional analysis resolves the long-standing dichotomy in PMWS pathology of whether or not apoptosis is implicated in lymphoid depletion in vivo [31–33]. In lymphoid tissue with low-level replication, it is not. On the other hand, in PMWS lymph nodes collapsing under PCV2, genes mediating apoptosis are in full force (Fig. 4).

Another example of PorSignDB generating intriguing hypotheses, is that weaned gut gene expression signatures are induced in clinical PCV2, while intestinal signatures of suckling piglets are suppressed (Additional file 4). This echoes the clinical observation that pigs are most susceptible to PMWS at time of weaning. It suggests that as long as intestinal tissue is protected by maternal antibodies, progression to PMWS is obstructed. On the other hand, when weaned, naive intestinal tissue makes immunological contact with pathogens, producing a microenvironment that reflects PMWS and hence, may promote PCV2.

When it comes to the PCV2 disease signature, caution should be applied. It suggests that activation of myeloid leukocytes, such as monocytes or macrophages, is a key element distinguishing PMWS pigs from subclinically infected pigs. However, for the generation of a valid molecular signature, it is necessary that the training and validation cohorts are similar. Even though these cohorts are highly comparable on a clinical level (i.e. pathological lesions such as viral load, degree of lymphoid depletion and granulomatous inflammation [11], also indicated in Additional file 9), no information is available on their co-infection status. It is possible that this disease signature represents a specific co-infection that was circulating in swine farms at the time, and that the use of this signature is therefore restricted to that particular co-infection. Whether the PCV2 disease signature is widely applicable thus remains to be confirmed in the future by other cohorts.

Finally, the pronounced IL-2 signature in clinical PCV2 inspired the establishment of primary lymphoblast strains. They can be easily expanded and stored in liquid nitrogen, and display excellent post-thaw survival. Unlike PK-15 cells, they can be harvested from different individuals or breeds, providing a new and valuable tool for studying the long-suspected impact of genetic background on PCV2 replication [34, 35]. However, a limitation of this cell culture system is that it does not fully recapitulate PMWS pathology. Upon IL-2 stimulation, cell death is prevented and mitosis upregulated (Fig. 5b). In contrast, in PMWS, cell cycle progression networks are downregulated (Fig. 2c, Fig. 4). The latter seems contradictory as PCV2 genome replication highly depends on host cell polymerases, and hence, cells in mitosis [36]. This can be explained by the fact that PMWS is an end-stage of disease, where fulminant PCV2 replication has already taken place, lymphoid parenchyma is overloaded with PCV2 particles, and germinal centers have collapsed. It also indicates that increasing the mitotic index is not sufficient for generating the fulminant replication leading to PMWS. Other factors are needed, and this study suggests that the activation of myeloid leukocyte mediated inflammatory host responses may be another element of the puzzle. In any case, whether the IL-2 cytokine itself is upregulated in PMWS lymph nodes has never been demonstrated. It is tempting to think that co-infections such as bacterial or viral pathogens cause an infusion of IL-2 in the lymph nodes, but this remains to be proved.

## Conclusion

In conclusion, we here suggest a model to understand how PCV2 establishes subclinical persistence, and how it switches to clinical disease. Upon infection, PCV2 replicates at modest rates which seem unable to trigger a powerful immune response. This may cause lymphoid tissue to act is if the pathogen is absent. Whenever an individual falls victim to a stimulus that rewires the transcriptional circuitry with a myeloid leukocyte mediated immune activation, PCV2 replicates frantically and overwhelms the host. Given its limited coding capacity, PCV2 cannot manage it alone but depends on superinfections to recalibrate the host. This may help to explain how PCV2 circulates in pig farms.

## Materials and methods

### Transcriptomic analysis

For transcriptomic studies, raw data were retrieved from NCBI GEO (http://www.ncbi.nlm.nih.gov/geo/). GEO accession numbers include GSE7313, GSE7314, GSE8974, GSE12705, GSE13528, GSE14643, GSE14758, GSE14790, GSE15211, GSE15256, GSE15472, GSE16348, GSE17264, GSE17492, GSE18343, GSE18359, GSE18467, GSE18641, GSE18854, GSE19083, GSE19275, GSE19975, GSE21043, GSE21071, GSE21096, GSE21383, GSE21663, GSE22165, GSE22487, GSE22596, GSE22782, GSE23503, GSE23596, GSE23751, GSE24239, GSE24762, GSE24889, GSE26095, GSE26663, GSE27000, GSE28003, GSE30874, GSE30956, GSE31191, GSE32956, GSE33037, GSE33246, GSE34569, GSE36306, GSE37166, GSE37922, GSE40885, GSE41636, GSE43072, GSE44326, GSE47710, GSE47814, GSE48125, GSE48839, GSE49290, GSE53997, GSE64246, GSE65008, GSE66317, GSE72025, GSE73088 and GSE106471. For microarray studies, quantile normalized expression data was generated from .CEL files using the ExpressionFileCreator module on Genepattern [37]. Affymetrix porcine genechip probe set identifiers were mapped to *Homo sapiens* gene symbols as previously described [38] with Refseq and Uniprot identifiers were changed into corresponding gene symbols. For Affymetrix HG-U133 plus 2, GSEA chip annotations were employed. For RNA-seq, SRA files were converted to Fastq files with Genepattern SraToFastQ module. Reads were mapped to *Mus musculus* mm10 genome assembly with Genepattern tophat module, and converted to normalized to RPKM read counts using cuffnorm on the galaxy public server [39]. GSEA analyses were performed with GSEA desktop v3.0 (http://software.broadinstitute.org/gsea/index.jsp).

### Generating PorSignDB

Affymetrix Porcine Genechip data available on NCBI GEO were curated as follows. Data covering pooled samples or lacking publication on Pubmed were discarded, as were studies with < 2 samples per phenotype. Early transcriptional responses (< 30 mins) and comparisons between breeds or tissue types were ignored. If controls were unavailable for temporal studies, comparisons were made with *t* = 0. For signature generation, the ImmuneSigDB recipe [13] was followed. Briefly, genes were correlated to a target profile and ranked using the RNMI metric [40]. Top and bottom ranked genes with an FDR < 0.01 in a permutation test were included in two gene sets, with minimally 100 and maximally 200 genes each, yielding “PHENOTYPE1_VS_PHENOTYPE2_UP” and “PHENOTYPE1_VS_PHENOTYPE2_DN”. To ensure informative gene set comparisons, a GO biological process term enrichment was performed for every gene set using clusterProfiler [41]. Comparisons where either UP or DN gene set yielded zero significant hits (*p* < 0.05, Benjamini-Hochberg corrected) were discarded.

### PCV2 disease signature and phenotype classification

Biomarker genes were calculated from data of a field study covering three different cohorts [11], according to a previously described method [42] with minor modifications. Cohorts were divided over a training set (*n* = 17) and a validation set (*n* = 8). Marker genes were ranked in the training set using signal-to-noise ratio (S2NR), with standard deviations adjusted to minimally 0.2*mean. In a subsequent leave-one-out cross validation, a single sample was left out and a permutation test was performed on the remaining samples. Only genes with p < 0.05 in every iterative leave-one-out trial were included in the signature. For phenotype classification, the Nearest Template Prediction (NTP) algorithm [20] was employed with S2NR as weights.

### Cells, virus and reagents

PCV1-negative PK15 (Porcine Kidney-15) cells were a kind gift of Gordon Allan, Queen’s University, Belfast, UK. PK15 culture conditions were described earlier [43]. To generate PPLs, PBMCs were isolated from whole blood collected from hybrid Pietrain x Hypor Libra pigs by density centrifugation as described previously [26]. After adhering of monocytes to a plastic culture flask, lymphocytes in suspension were pelleted, resuspended in culture medium supplemented with 5 μg/ml ConA (Sigma) and 50 μM β-mercaptoethanol (Gibco). After three days, cells were pelleted, washed with RPMI (Gibco), and resuspended in culture medium supplemented with 100 U/ml human recombinant IL-2 (NIH) and 50 μM β-mercaptoethanol. Porcine alveolar macrophages were isolated as described [44]. PCV2 strains 1121 and Stoon1010 were described previously [45]. PRRSV Lelystad virus strain (LV) was described earlier [44].

### Experimental infection and immunostaining

PK-15 and PPLs were inoculated with PCV2 1121 at 0.1 TCID_50_/cell for 1 h, washed and further incubated in culture medium for 36 h. For Cpd188 experiments, cells were pre-incubated for 1 h with Cpd188 (Merck Millipore) dissolved in 0.25% DMSO. Subsequently, cells were inoculated with PCV2 1121 at 0.1 TCID_50_/cell for 1 h, washed and incubated for 72 h. For co-culture, PPLs and macrophages were inoculated at 0.5 TCID_50_/cell for 1 h with PCV2 Stoon1010 and PRRSV respectively, washed and incubated for 72 h. PCV2 capsid immunostaining with monoclonal antibody (mAb) 38C1 was described earlier [43].

For showing that PPLs are not susceptible to PRRSV, Lymphoblasts were incubated with PRRSV LV strain at a MOI of 0.5 or with media at 37C. After 1 h, the inoculum/media was removed and cells were further cultured for 72 h. Cells were stained with a mouse mAb 13E2 against nucleocapsid protein (produced in our lab, 1/50) [46], followed by an FITC-conjugated goat-anti-mouse IgG antibody (1/200; Invitrogen). Cell nuclei were counterstained with Hoechst 33342 (1/100; Invitrogen). All cell visualizations were performed with TCS SPE confocal system (Leica Microsystems GmbH, Germany). Alveolar macrophages were inoculated with PRRSV LV strain and immunostained as a positive control.

## Additional files

Additional file 1: GO biological process term enrichment of every gene set in PorSignDB. (XLSX 662 kb)

Additional file 2: Overlap of bacterial and viral gene sets in PorSignDB. (PDF 63 kb)

Additional file 3: Overlap of *Salmonella* Typhimurium and *Salmonella* Choleraesuis gene sets in PorSignDB. (PDF 36 kb)

Additional file 4: PorSignDB signatures as HUGO gene symbols, in gmt format. (GMT 344 kb)

Additional file 5: PorSignDB signatures as entrez symbols, in gmt format. (GMT 305 kb)

Additional file 6: PorSignDB performance in lymph nodes of PMWS pigs VS healthy pigs. Figure displays enriched PorSignDB gene sets in the PMWS study pertaining to biological themes other than microbiology. (PDF 92 kb)

Additional file 7: Complete list of PCV2 disease signature biomarker genes. (XLSX 15 kb)

Additional file 8: PMWS biomarker genes annotation and performance of an alternative clinical disease signature. **A** Gene ontology (GO) terms overrepresentation test of PMWS biomarker genes. **B** Nearest Template Prediction of test set samples using an alternative clincal gene signature based on the RNMI metric **C** and similarly, of the experimental subclinical infection samples at 29dpi. (ZIP 121 kb)

Additional file 9: STAT3 is a host factor in PCV2 disease. **A** Core genes responsible for the STAT3 signature enrichment score. **B** STAT3-specific inhibitor cpd-188 impairs infection in PK-15 cells. Means ± sd represents three independent experiments in triplicate (*n* = 9; **P < 0.01, ***P < 0.001, Mann-Whitney U-Test). **C** MTT cell viability assay of cpd-188 treatment in PK-15 cells. Means ± sd are shown for three independent experiments in quintuplicate (*n* = 15). **D** Infection assessment by PCV2 capsid immunostaining, representative figures for each treatment. Scale bar: 100 μm. (PDF 162 kb)

Additional file 10: ImmuneSigDB analysis of the PMWS field study dataset. (XLSX 16 kb)

Additional file 11: PRRSV infection trial of primary porcine lymphoblasts. (PDF 145 kb)

Additional file 12: PorSignDB annotation source file. (XLSX 25 kb)

## Abbreviations

ConA: Concanavalin A
Dpi: Days post inoculation/ days post infection
FDR: False discovery rate
GO: Gene ontology
GSEA: Gene set enrichment analysis
Hpi: Hours post inoculation
IFNα: Interferon alfa
IFNγ: Interferon gamma
IL-2: Interleukin 2
IRF3: Interferon regulatory factor 3
JAK: Janus kinase
KRas: Kirsten rat sarcoma
LPS: Lipopolysaccharide
LV: PRRSV Lelystad virus strain
mAb: Monoclonal antibody
NF-κB: Nuclear Factor Kappa B
NTP: Nearest template prediction
ORF: Open reading frame
PBMC: Peripheral blood monocytic cell
PCV2: Porcine circovirus type 2
PMWS: Postweaning multisystemic wasting syndrome
PPL: Primary porcine lymphoblast
PRRSV: Porcine reproductive and respiratory Syndrome virus
RNMI: Rescaled normalized mutual information
ROS: Reactive oxygen species
STAT3: Signal transducer and activator of transcription 3
STAT5: Signal transducer and activator of transcription 5
TCID50: Tissue culture infectious dose 50
Wt: Wild type
