# Supplementary material for: Dissecting clinical outcome of porcine circovirus type 2 with in vivo derived transcriptomic signatures of host tissue responses
Source: BMC Genomics. 2018 Nov 20;19:831. doi: 10.1186/s12864-018-5217-5 (PMC6247532; doi:10.1186/s12864-018-5217-5)
Supplement: Supplementary file 2 — Overlap of bacterial and viral gene sets in PorSignDB. (PDF 63 kb) [file 12864_2018_5217_MOESM2_ESM.pdf]

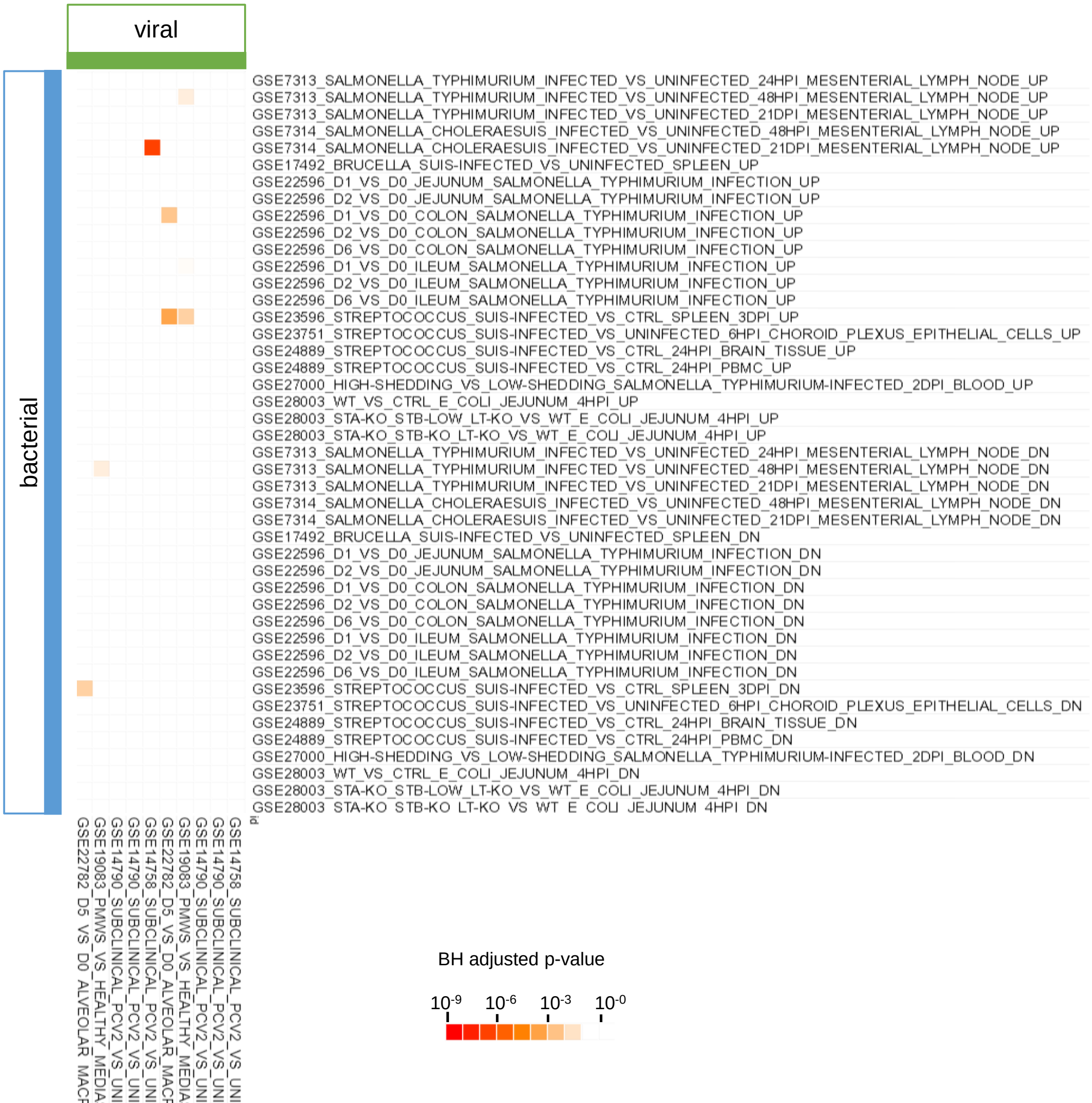


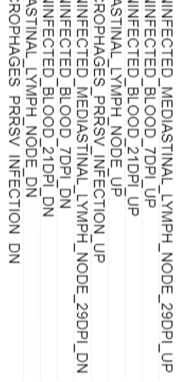

**Additional file 2:** Gene set overlap of bacterial and viral gene sets in PorSignDB. In order to see whether bacterial gene sets differed from bacterial gene sets, overlap of genes was calculated using a hypergeometric test with Benjamini-Hochberg (BH) adjusted p-values.
