# Supplementary material for: Dissecting clinical outcome of porcine circovirus type 2 with in vivo derived transcriptomic signatures of host tissue responses
Source: BMC Genomics. 2018 Nov 20;19:831. doi: 10.1186/s12864-018-5217-5 (PMC6247532; doi:10.1186/s12864-018-5217-5)
Supplement: Supplementary file 6 — PorSignDB performance in lymph nodes of PMWS pigs VS healthy pigs. Figure displays enriched PorSignDB gene sets in the PMWS study pertaining to biological themes other than microbiology. (PDF 92 kb) [file 12864_2018_5217_MOESM6_ESM.pdf]

PorSignDB performance in lymph nodes of PMWS pigs VS healthy pigs

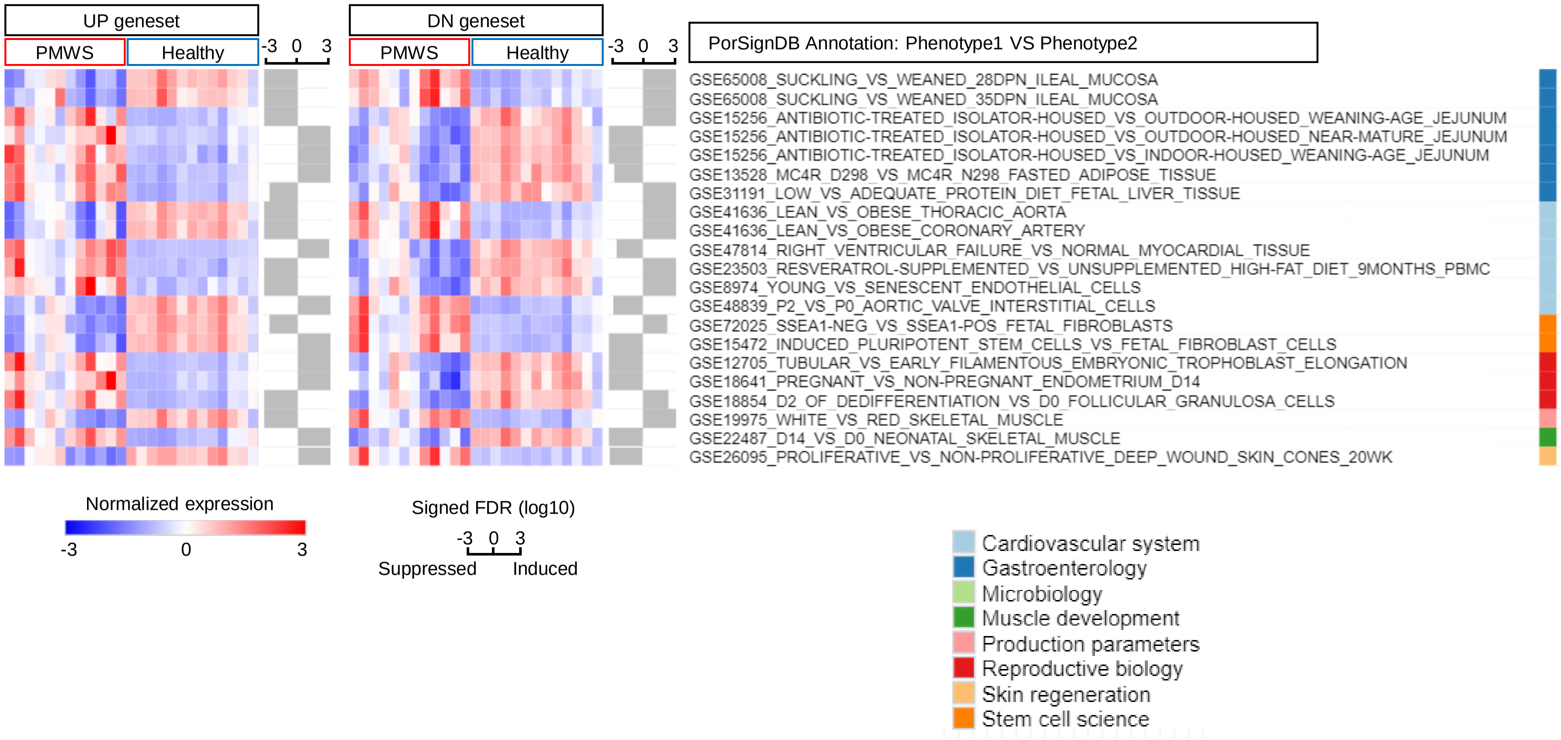

Additional file 6: Enriched PorSignDB gene sets in the PMWS field study pertaining to biological themes other than microbiology. The average expression of the leading-edge genes in every gene set (genes that contribute to the enrichment) are displayed for each patient sample. Bars next to each gene set indicate the signed FDR for its enrichment in log10 scale.
