# Supplementary material for: Dissecting clinical outcome of porcine circovirus type 2 with in vivo derived transcriptomic signatures of host tissue responses
Source: BMC Genomics. 2018 Nov 20;19:831. doi: 10.1186/s12864-018-5217-5 (PMC6247532; doi:10.1186/s12864-018-5217-5)
Supplement: Supplementary file 8 — PMWS biomarker genes annotation and performance of an alternative clinical disease signature. A Gene ontology (GO) terms overrepresentation test of PMWS biomarker genes. B Nearest Template Prediction of test set samples using an alternative clincal gene signature based on the RNMI metric C and similarly, of the experimental subclinical infection samples at 29dpi. (ZIP 121 kb) [file 12864_2018_5217_MOESM8_ESM.zip › additional-file8.pptx]

## Slide 1
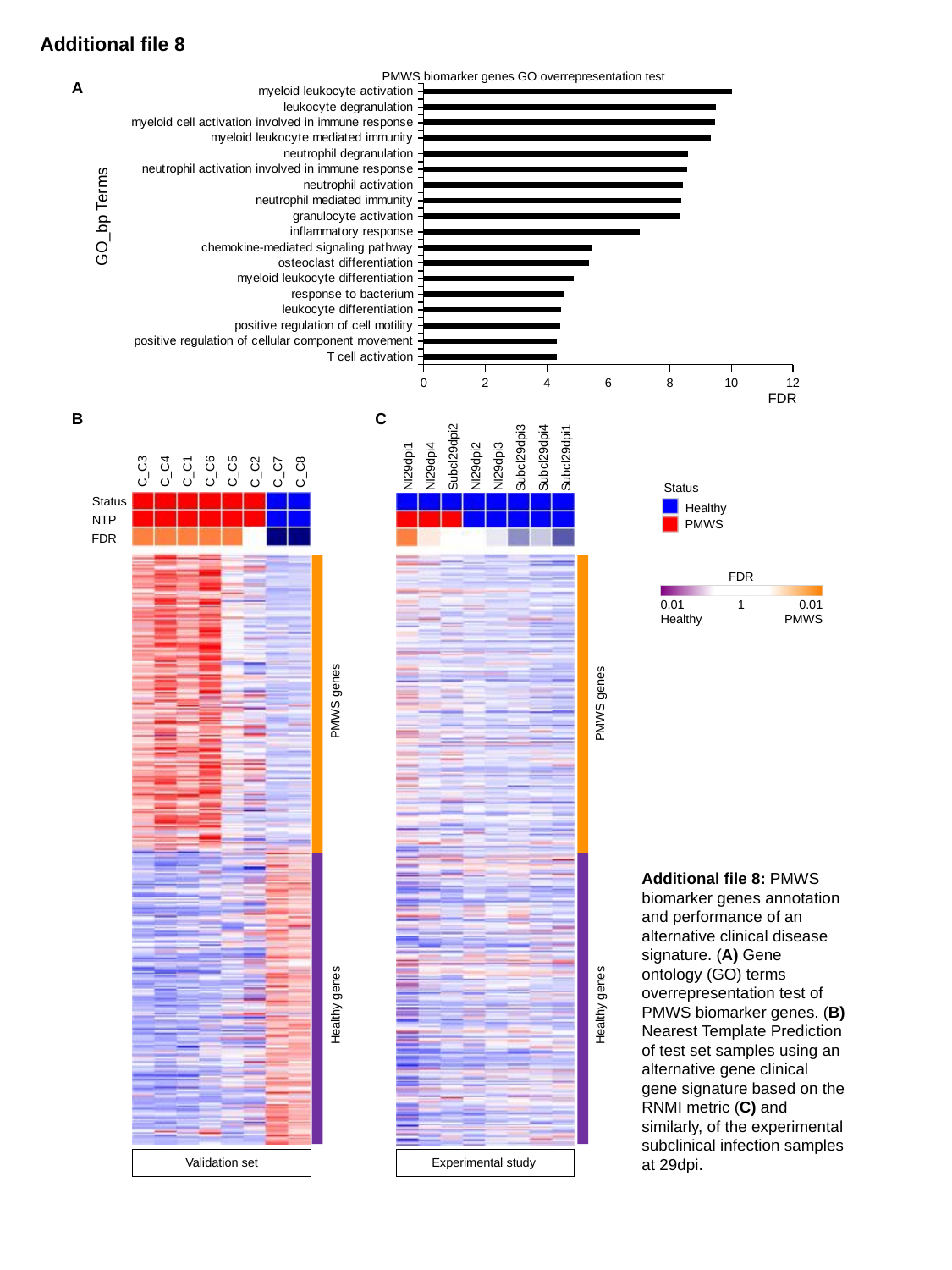

Additional file 8
PMWS biomarker genes GO overrepresentation test
A
### Chart
| Category | |
|---|---|
| T cell activation | 4.320484445084266 |
| positive regulation of cellular component movement | 4.326633579226203 |
| positive regulation of cell motility | 4.4280900926377535 |
| leukocyte differentiation | 4.466917661648882 |
| response to bacterium | 4.572620009474286 |
| myeloid leukocyte differentiation | 4.888262742293094 |
| osteoclast differentiation | 5.364092758639169 |
| chemokine-mediated signaling pathway | 5.447979192243934 |
| inflammatory response | 7.011976379157448 |
| granulocyte activation | 8.343979976839872 |
| neutrophil mediated immunity | 8.362759984995396 |
| neutrophil activation | 8.438485318079778 |
| neutrophil activation involved in immune response | 8.573386351912003 |
| neutrophil degranulation | 8.59291085103852 |
| myeloid leukocyte mediated immunity | 9.326110199680953 |
| myeloid cell activation involved in immune response | 9.461443272440688 |
| leukocyte degranulation | 9.500626202771496 |
| myeloid leukocyte activation | 10.017796111715423 |GO_bp Terms
FDR
B
C
Subcl29dpi1
Subcl29dpi3
Subcl29dpi4
Subcl29dpi2
NI29dpi1
NI29dpi4
NI29dpi2
NI29dpi3
PMWS genes
Healthy genes
Experimental study
C_C3
C_C4
C_C1
C_C2
C_C7
C_C8
C_C6
C_C5
Status
NTP
FDR
Validation set
PMWS genes
Healthy genes
Status
Healthy
PMWS
FDR
0.01
Healthy
1
0.01
PMWS
Additional file 8: PMWS biomarker genes annotation and performance of an alternative clinical disease signature. (A) Gene ontology (GO) terms overrepresentation test of PMWS biomarker genes. (B) Nearest Template Prediction of test set samples using an alternative gene clinical gene signature based on the RNMI metric (C) and similarly, of the experimental subclinical infection samples at 29dpi.
