# Supplementary figures and images for: Dissecting clinical outcome of porcine circovirus type 2 with in vivo derived transcriptomic signatures of host tissue responses
Source: BMC Genomics. 2018 Nov 20;19:831. doi: 10.1186/s12864-018-5217-5 (PMC6247532; doi:10.1186/s12864-018-5217-5)

### Additional file 8

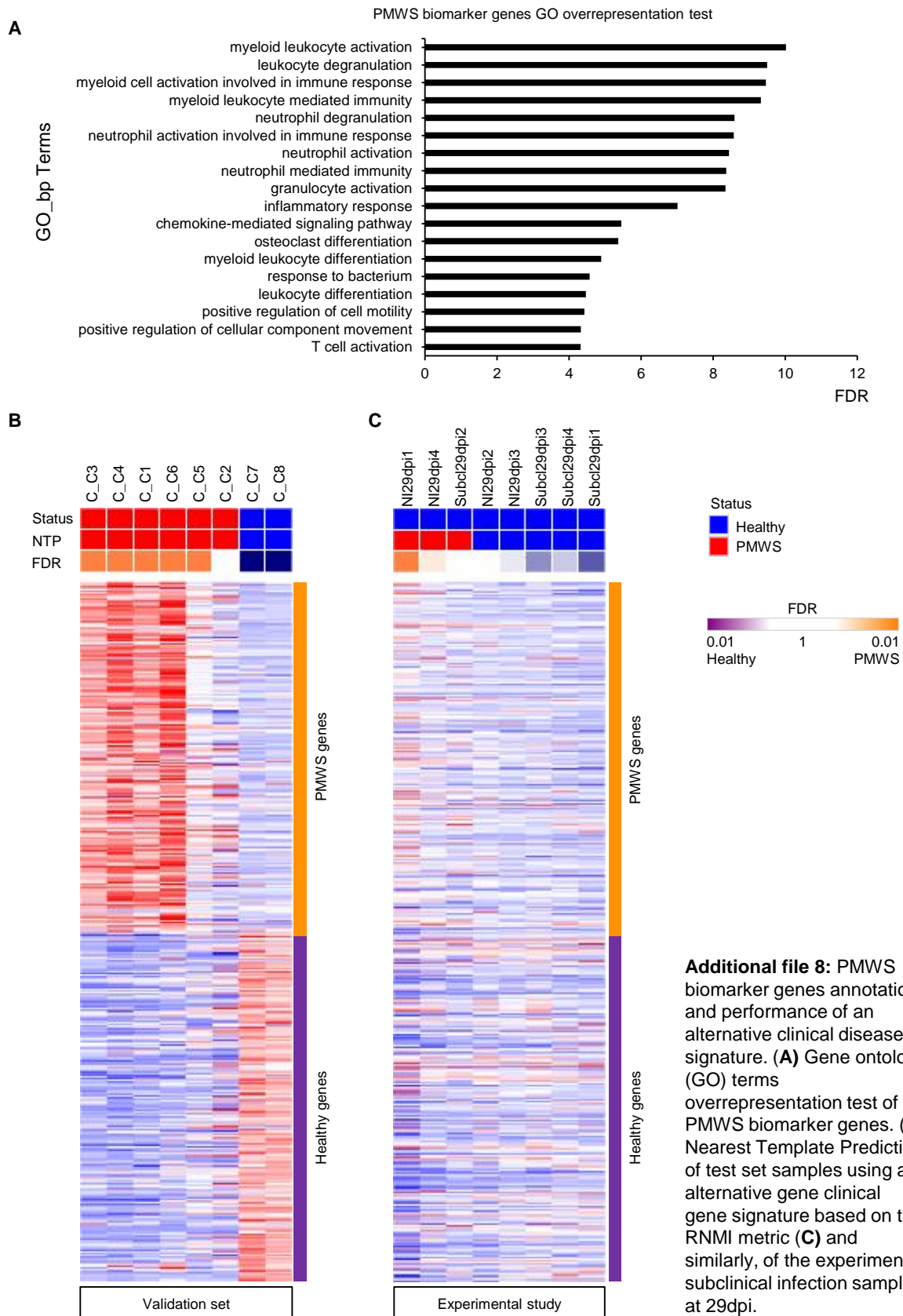

Supplement: Supplementary file 8 — PMWS biomarker genes annotation and performance of an alternative clinical disease signature. A Gene ontology (GO) terms overrepresentation test of PMWS biomarker genes. B Nearest Template Prediction of test set samples using an alternative clincal gene signature based on the RNMI metric C and similarly, of the experimental subclinical infection samples at 29dpi. (ZIP 121 kb) [file 12864_2018_5217_MOESM8_ESM.zip › additional-file8.pdf]
