# Supplementary material for: Dissecting clinical outcome of porcine circovirus type 2 with in vivo derived transcriptomic signatures of host tissue responses
Source: BMC Genomics. 2018 Nov 20;19:831. doi: 10.1186/s12864-018-5217-5 (PMC6247532; doi:10.1186/s12864-018-5217-5)
Supplement: Supplementary file 9 — STAT3 is a host factor in PCV2 disease. A Core genes responsible for the STAT3 signature enrichment score. B STAT3-specific inhibitor cpd-188 impairs infection in PK-15 cells. Means ± sd represents three independent experiments in triplicate (n = 9; **P < 0.01, ***P < 0.001, Mann-Whitney U-Test). C MTT cell viability assay of cpd-188 treatment in PK-15 cells. Means ± sd are shown for three independent experiments in quintuplicate (n = 15). D Infection assessment by PCV2 capsid immunostaining, representative figures for each treatment. Scale bar: 100 μm. (PDF 162 kb) [file 12864_2018_5217_MOESM9_ESM.pdf]

Additional file 9

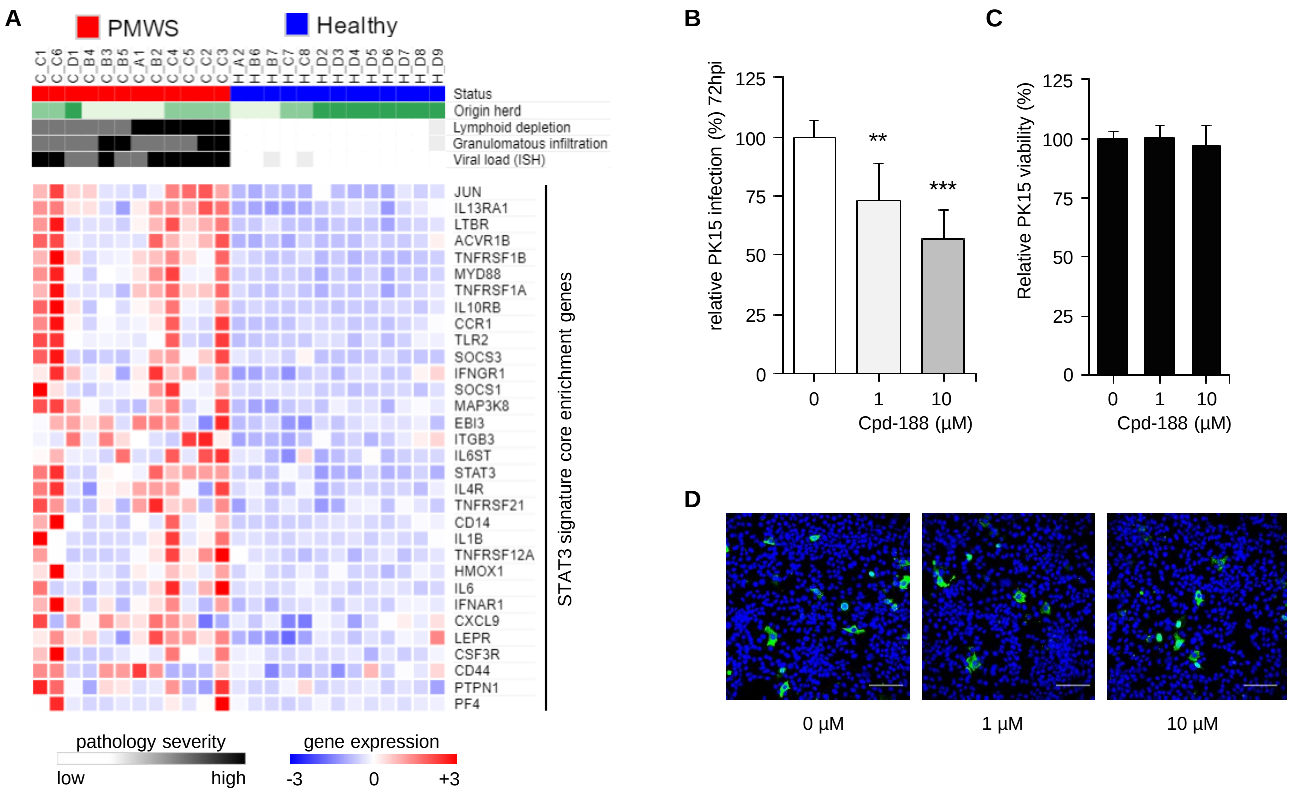

Additional file 9: STAT3 as a host factor in PCV2 disease **A** Core genes responsible for the STAT3 signature enrichment score. **B** STAT3-specific inhibitor cpd-188 impairs infection in PK-15 cells. Means ± sd represent three independent experiments in triplicate (n=9; **P<0.01, ***P<0.001, Mann-Whitney U test). **C** MTT viability assay of cpd-188 treatment in PK-15 cells. Means ± sd are shown for three independent experiments in quintuplicate (n=15). **D** Infection assessment by PCV2 capsid immunostaining, representative figures for each treatment. Scale bar: 100 µm.
