# Supplementary material for: Dissecting clinical outcome of porcine circovirus type 2 with in vivo derived transcriptomic signatures of host tissue responses
Source: BMC Genomics. 2018 Nov 20;19:831. doi: 10.1186/s12864-018-5217-5 (PMC6247532; doi:10.1186/s12864-018-5217-5)
Supplement: Supplementary file 11 — PRRSV infection trial of primary porcine lymphoblasts. (PDF 145 kb) [file 12864_2018_5217_MOESM11_ESM.pdf]

Additional file 11

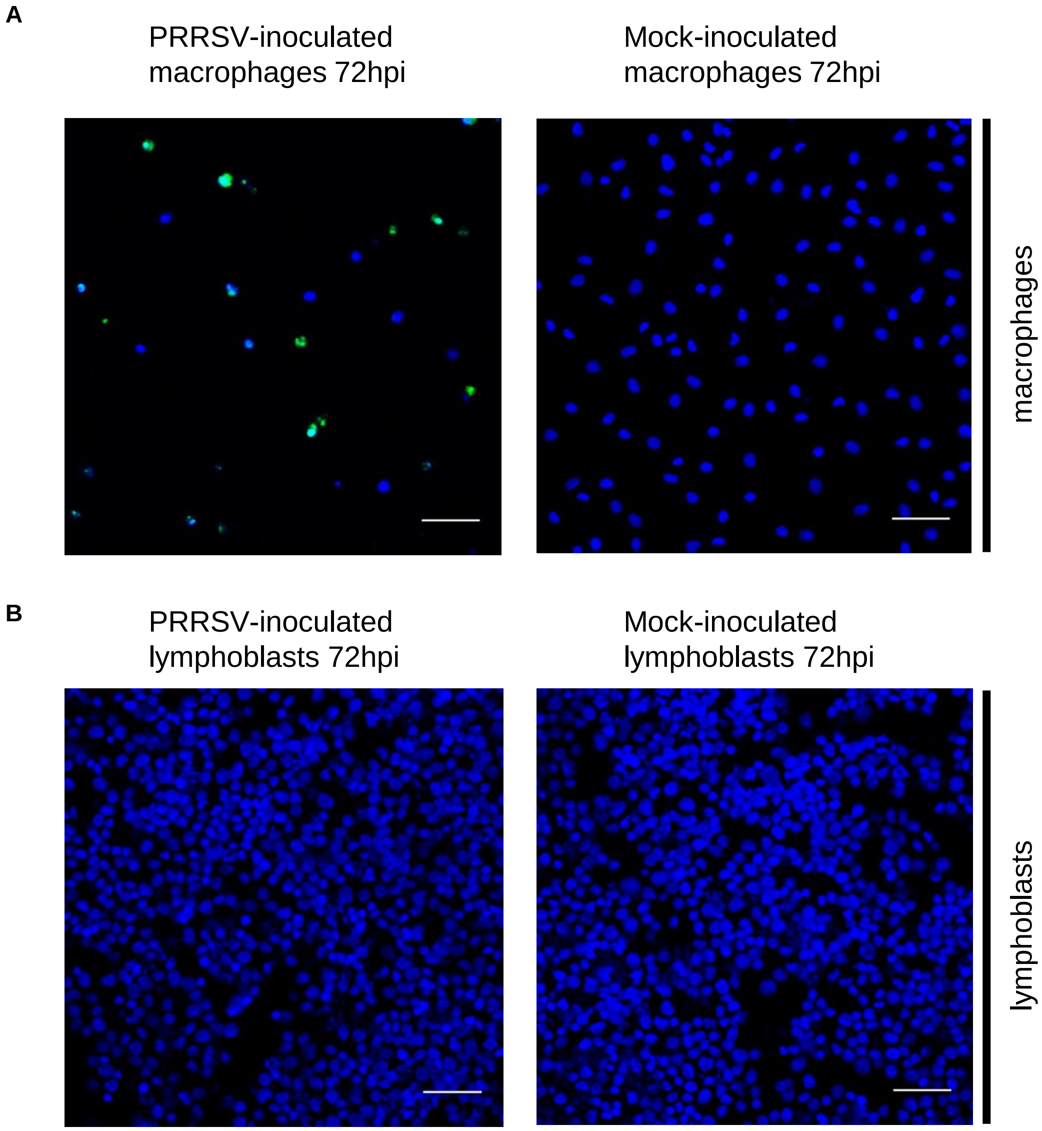

Additional file 11: Lymphoblasts do not support PRRSV replication **A** Primary porcine alveolar macrophages were infected with PRRSV strain LV, or mock-infected. PRRSV nucleocapsid was stained through immunofluorescence. Given PRRSV's narrow tropism for macrophages, macrophages are permissive for PRRSV and show a positive signal for PRRSV nucleocapsid. Mock-inoculated macrophages serve as a negative control. **B** Similarly, primary porcine lymphoblasts were infected by PRRSV strain LV, or mock-infected and stained for PRRSV nucleocapsid. As expected, both were negative for PRRSV, confirming that primary porcine lymphoblasts are not susceptible to PRRSV replication. Scale bar: 50 µm.
